# Exploring Nursing Implications in the Standards of Care for Transgender and Gender Diverse Health, Version 8 by Coleman et al. (2022)

**DOI:** 10.1111/jan.16638

**Published:** 2024-11-19

**Authors:** John Gilmore

**Affiliations:** ^1^ School of Nursing Midwifery and Health Systems, University College Dublin Dublin Ireland

## 1 Introduction

There is more awareness and focus than ever before on issues of the health and healthcare needs of gender minority groups such as transgender and gender diverse communities (TGD) and with that comes a responsibility for nurses to develop the knowledge and skills required for effective and appropriate care (Gilmore, Dainton, and Halpin 2024). The World Professional Assocation for Transgender Health (WPATH) Standards of Care (SOC‐8) guidelines (Coleman et al. 2022) provide the most substantial guide for the provision of care to TGD communities globally. WPATH, or the World Professional Association for Transgender Health, is an international multidisciplinary organisation composed of healthcare professionals, social scientists and transgender rights advocates dedicated to advancing the health and well‐being of TGD individuals.

The SOC‐8 mark a comprehensive advancement in healthcare for TGD individuals, addressing a wide array of healthcare needs across multiple disciplines, settings and life stages. For nursing and healthcare professionals, these guidelines offer an in‐depth framework aimed at providing respectful, evidence‐based and inclusive care, which is essential for promoting the holistic well‐being of TGD individuals. The SOC‐8 guidelines expand upon previous standards by encompassing not only the physical health needs of TGD patients but also the social, psychological and developmental factors that influence their healthcare experiences and outcomes.

## 2 TGD Health Inequalities

Studies reveal that TGD communities experience heightened vulnerability to physical and mental health disparities due to systemic barriers, discrimination and stigma in healthcare settings. Zeeman et al. (2019) emphasise that these health inequalities are exacerbated by a lack of provider knowledge, implicit biases and inadequate healthcare infrastructure tailored to TGD needs. This often results in TGD individuals facing higher rates of untreated chronic conditions, increased mental health challenges such as depression and anxiety, and an overall diminished trust in healthcare systems. Drabish and Theeke (2022) similarly underscore the impact of social exclusion and minority stress on the well‐being of TGD populations, linking these factors to elevated rates of self‐harm, substance use and suicidality. These findings support the necessity of SOC‐8's focus on inclusive, gender‐affirming and harm‐reduction approaches, urging healthcare providers to actively work to reduce these health disparities by creating safe, knowledgeable and inclusive environments for TGD patients.

## 3 Methods

A multi‐stage process for the development of the guidelines is outlined, designed to ensure the recommendations are evidence‐based, contextually appropriate and clinically actionable. The initial stages listed involved reviewing topics from the previous SOC‐7 guidelines and identifying key questions with input from an Evidence Review Team, a Guideline Steering Committee and WPATH Chapter Leads. Systematic literature reviews were conducted for areas requiring robust evidence, and recommendation statements were drafted based on these findings. Chapter leads then refined these statements through detailed discussion with co‐authors, ensuring alignment with current research and clinical expertise.

A Delphi method was employed to achieve consensus on each recommendation statement. Using a scale from 1 (strongly disagree) to 9 (strongly agree), each recommendation needed at least 75% agreement to be approved. Approved statements were then graded using the GRADE framework to determine the strength of each recommendation, with ‘strong’ recommendations for high‐quality evidence and broad patient acceptance and ‘weak’ recommendations where evidence was less certain or patient acceptance might vary, certainty of evidence ratings (+ to ++++) were appended to statements supported by systematic literature reviews, and a final level of agreement was provided in the appendix.

## 4 Guideline Contents

The guidelines span 18 chapters, each addressing distinct areas related to health or healthcare. Foregrounded by a chapter on Terminology, it should be noted that the term TGD is used throughout the guidelines; however, the authors acknowledge that this is not universally preferred language. Recognising that culturally sensitive terminology promotes safety, dignity and respect in healthcare, the guidelines recommend healthcare providers discuss and honour preferred language with TGD individuals to support a respectful, affirming healthcare environment (Coleman et al. 2022).

An acknowledged limitation of the guidelines is that the research underpinning them is overwhelmingly from a North American and European perspective. The authors emphasise that TGD care should not be constrained by Western‐centric practices but instead adaptable to regional sociocultural and political contexts to make the care accessible and meaningful worldwide (Coleman et al. 2022). This flexibility is particularly valuable for healthcare professionals in various regions who may face different barriers and resources in providing TGD care. While the provision of equitable and appropriate gender‐affirming healthcare services as a right is central to the SOC‐8, the intersection of legal and wider social rights for TGD communities cannot be underestimated (Gilmore 2023).

The education chapter is particularly important for nurses and healthcare professionals as it underscores the need for comprehensive training on TGD health to reduce knowledge gaps and biases. It emphasises that healthcare providers must be equipped with competencies in TGD care to prevent situations where TGD patients have to educate providers about their healthcare needs (Coleman et al. 2022). There remain significant gaps in nursing education around the issue of sexuality and gender diversity, and a concerted systematic effort should be encouraged to including issues of Lesbian, Gay, Bisexual, Transgender, Queer, Intersex (LGBTQI)+ health into the curriculum (McCann and Brown 2018). Enhanced education and training can foster a more inclusive environment, reduce stigma and improve the quality of care TGD individuals receive.

The SOC‐8 guidelines also address assessment processes for both adults and adolescents, acknowledging the exponential increase in referrals for TGD care and the unique developmental considerations of younger populations. For healthcare professionals, understanding these distinctions is critical in providing age‐appropriate, individualised care that respects the developmental stages of TGD adolescents while also supporting informed decision‐making among adults. The fundamental principle of informed consent as a basis for gender‐affirming healthcare is clearly foreground, but the complexities around family‐centred and parental inclusion are key considerations when caring for adolescents seeking this type of medical treatment.

The Hormone Therapy and Surgery and Postoperative Care chapters provide detailed clinical guidelines for initiating and managing gender‐affirming treatments, emphasising a patient‐centred, multidisciplinary approach. These chapters detail a variety of medical and surgical interventions and underscore that there is no one‐size‐fits‐all model for TGD care, recognising that each individual's journey and needs are unique. Healthcare professionals are encouraged to work closely with patients, respecting their personal goals while providing safe and effective care pathways.

The guidelines also expand upon clinical care in various settings, including Primary Care, Reproductive Health, Mental Health and Sexual Health. Each of these areas underscores the importance of multidisciplinary, holistic support for TGD patients. For example, the Primary Care chapter encourages routine preventive care, such as cancer screenings, tailored to TGD individuals, while Reproductive Health covers fertility preservation and contraceptive needs in gender‐diverse populations. Language is central to ensuring more affirming and respectful care, and in highly gendered settings, such as maternal and reproductive healthcare this can be even more sensitive (Pezaro et al. 2024). The chapter on sexual health highlights the importance of sexual satisfaction and safety, advocating for TGD people's right to fulfilling sexual lives. These recommendations ensure that healthcare providers offer continuity of care, have high standards across the life course and health system, as well as to address chronic health needs, and promote preventive measures in TGD populations. There is also now a chapter on Voice and Communication which focuses beyond traditional medical or surgical treatments, where interventions such as voice therapy is framed to affirm communication styles beyond binary expressions of gender.

A notable gap in the SOC‐8 guidelines is the absence of specific recommendations for the care of TGD individuals in acute and critical care settings. Managing these patients in high‐acuity environments requires attention to several unique considerations, including airway management (due to facial surgeries), the effects of cross‐sex hormones on biochemistry and inotropic responses can influence treatment decisions and pharmacological choices, while the elevated risk of venous thromboembolism (VTE), particularly among those on oestrogen therapy, necessitates careful risk assessment and anticoagulation planning (Gilmore, Dainton, and McEvoy 2024). These complexities highlight the need for further research and guidelines to ensure safe, competent and inclusive care for TGD patients in acute and critical care contexts.

Of particular importance is the chapter on Mental Health, which outlines a patient‐centred approach that emphasises harm reduction and rejects mandatory psychotherapy for access to gender‐affirming care. It urges providers to consider the impacts of societal discrimination, advocating against conversion therapies and instead supporting mental health services that affirm the identities of TGD individuals (Coleman et al. 2022). Addressing mental health with a focus on inclusivity and respect is essential for healthcare providers, as minority stress due to societal stigma disproportionately affects the TGD community (Zeeman et al. 2019).

In response to systemic barriers and the sometimes fragmented care available to TGD individuals, SOC‐8 highlights the need for harm‐reduction approaches in cases where patients may seek non‐prescribed hormones due to lack of access (Coleman et al. 2022). Healthcare providers are encouraged to help mitigate these risks by offering safe, medically supervised alternatives where possible. This approach acknowledges healthcare disparities and empowers professionals to work towards more equitable access, even in resource‐limited settings. The chapter on Institutional Environments, such as correctional facilities, clearly asserts that these settings must provide gender‐affirming care despite constraints.

The chapters on Nonbinary and Eunuch populations are an addition to the new guidelines, and along with the Intersex chapter, emphasise the diversity within TGD communities, urging healthcare providers to approach gender diversity with a nuanced understanding that avoids pathologising or stigmatising non‐traditional gender expressions.

## 5 Conclusion

In conclusion, SOC‐8 guidelines represent a progressive step in TGD healthcare, advocating for flexible, individualised and inclusive care practices. For nursing and healthcare professionals, adopting these guidelines means recognising TGD patients as whole individuals with diverse needs and ensuring that their care is responsive, respectful and based on the latest scientific evidence.

The approach taken to the development of SOC‐8 underscores a commitment to inclusivity and collaboration, with the guidelines shaped not only by clinicians and researchers but also by the essential insights and voices of TGD community representatives. This inclusive approach ensures that the standards reflect both scientific evidence and the lived experiences of TGD individuals, addressing real‐world needs and challenges faced by these communities. By actively engaging TGD advocates and representatives throughout the development process, SOC‐8 embodies a patient‐centred and respectful framework that prioritises the dignity, safety and holistic well‐being of TGD individuals. This collaboration strengthens the guidelines' relevance, fostering trust and advancing equity within healthcare settings worldwide, making SOC‐8 a robust, adaptable guide for all who provide care to TGD populations.

SOC‐8 not only outlines clinical standards but also challenges healthcare systems and providers to combat stigma, support policy changes and champion TGD‐inclusive healthcare practices, creating a foundation for improved health outcomes and equity for TGD communities globally. The adoption and implementation of WPATH SOC‐8 guidelines within nursing settings demonstrates a commitment to human rights, equitable care and social justice, and is a clear signifier of authentic allyship to TGD communities.

## Conflicts of Interest

The author is a member of PATHI Professional Association for Trans Health Ireland but this is not an affiliate group of WPATH. No financial support was provided for the writing of this article.

## Data Availability

No data were generated or utilised for this commentary, and no fieldwork permissions were required.
